# LED Illumination Spectrum Manipulation for Increasing the Yield of Sweet Basil (*Ocimum basilicum* L.)

**DOI:** 10.3390/plants10020344

**Published:** 2021-02-11

**Authors:** Md Momtazur Rahman, Mikhail Vasiliev, Kamal Alameh

**Affiliations:** School of Science, Edith Cowan University, 270 Joondalup Drive, Joondalup, WA 6027, Australia; vasiliev.mikhail@gmail.com (M.V.); kalameh@bigpond.net.au (K.A.)

**Keywords:** artificial lighting, energy use efficiency, protected horticulture, light exposure, far-red illumination, medicinal plants, water use efficiency, growth analysis

## Abstract

Manipulation of the LED illumination spectrum can enhance plant growth rate and development in grow tents. We report on the identification of the illumination spectrum required to significantly enhance the growth rate of sweet basil (*Ocimum basilicum* L.) plants in grow tent environments by controlling the LED wavebands illuminating the plants. Since the optimal illumination spectrum depends on the plant type, this work focuses on identifying the illumination spectrum that achieves significant basil biomass improvement compared to improvements reported in prior studies. To be able to optimize the illumination spectrum, several steps must be achieved, namely, understanding plant biology, conducting several trial-and-error experiments, iteratively refining experimental conditions, and undertaking accurate statistical analyses. In this study, basil plants are grown in three grow tents with three LED illumination treatments, namely, only white LED illumination (denoted W*), the combination of red (R) and blue (B) LED illumination (denoted BR*) (relative red (R) and blue (B) intensities are 84% and 16%, respectively) and a combination of red (R), blue (B) and far-red (F) LED illumination (denoted BRF*) (relative red (R), blue (B) and far-red (F) intensities are 79%, 11%, and 10%, respectively). The photosynthetic photon flux density (PPFD) was set at 155 µmol m^−2^ s^−1^ for all illumination treatments, and the photoperiod was 20 h per day. Experimental results show that a combination of blue (B), red (R), and far-red (F) LED illumination leads to a one-fold increase in the yield of a sweet basil plant in comparison with only white LED illumination (W*). On the other hand, the use of blue (B) and red (R) LED illumination results in a half-fold increase in plant yield. Understanding the effects of LED illumination spectrum on the growth of plant sweet basil plants through basic horticulture research enables farmers to significantly improve their production yield, thus food security and profitability.

## 1. Introduction

Global food demand is expected to increase by approximately 70 percent by 2050 due to increasing population growth [1]. The use of energy-efficient light-emitting diode (LED) sources in a protected-crop environment is an attractive approach that enables high-quality crops [2,3,4,5] to be produced cost-effectively, meeting human food demands. While white LED-generated photons can stimulate the plant photosynthesis process, the entire spectral components of white LED light would not equally participate in the photosynthesis process [6], and the impact of far-red wavelengths on the growth of sweet basil is not fully understood yet.

According to the Australian Department of Agriculture, horticulture production has the biggest market share in the Australian agriculture market, estimated to increase by 3% to $11.7 billion in 2020 [7]. Due to recurrent devastating natural calamities, such as bushfires, rainfall deficiency, Australian crop production has severely been affected, and this has encouraged farmers to adopt protected cropping practices to offer high-quality crops to consumers while maintaining a high level of food security by increasing consumer access to non-seasonal foods all over the year [8]. Hence, protected cropping systems used in urban areas have become one of the fastest-growing and dominant food-producing sector in Australian Horticulture, valued at around $1.8 billion per year in 2019, which is equivalent to 20% of the value of leafy vegetable food production in Australia [9]. Note that protected cropping offers several advantages, including diverse production structures (e.g., optical illumination in dark tents, greenhouses, vertical farms, etc.) [10], ambient temperature control, which maintains a high crop yield [11], better control of the CO_2_ [12] concentration, water, minerals, and fertilizers, which improve the photosynthesis process. The heating/cooling and lighting costs together typically stand for 25~35% of the total cost in a greenhouse environment [13]. Due to higher operation costs, conventional greenhouses are still unable to provide agricultural products to consumers cost-effectively. For example, sweet basil, parsley, coriander, and kale species are relatively expensive to produce commercially and require accurate adjustment of the growth conditions. These high costs have recently driven the market of LED-based indoor farming, mainly because of the high-efficiency, durability (~50,000 h), low-heat-generation and low-cost of light-emitting diode (LED) technology and the wide range of LED wavelength bands availability. LEDs are 40~70% more efficient than high-pressure sodium (HPS) lights or metal halide (MH) lamps (most common light sources used in indoor farming [13]).

**Figure 1:**
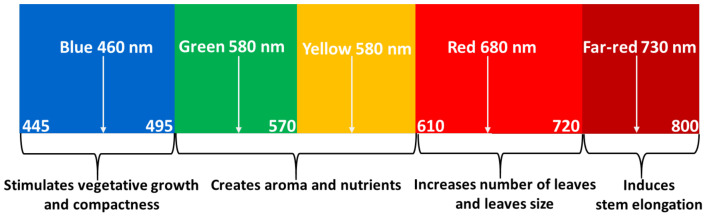
Effective wavelength bands that affect the growth of sweet basil plants.

Figure 1 shows the prominent wavelength bands that contribute to the development of sweet basil.

In addition, with the absence of sunlight at night times, LEDs can be used to illuminate the crop for a more extended period, thus shortening the crop cultivation cycle and improving the crop yield. In addition, LED-based high-insulation grow tents are relatively cheap and portable structures that can maintain ideal growing temperature and provide sufficient lighting at any time of day or night, in comparison with a greenhouse environment, where natural outdoor weather conditions dictate the cooling and heating requirements with a high degree of unpredictability. The grow tents were silver-coated on the inside and black on the outside. The grow tents were considerably remained closed for most of the time, except for panels with getting to permit airflow in, on three of four sides towards the tent’s base for ventilation. The fourth side was the tent entrance, and the zip was left open towards the bottom further to increase airflow and ventilation [14]. The tents were opened daily to water each plant; otherwise, the tents remained nearly closed. The visible light is typically the major contributor to the photosynthesis process for sweet basil plants [15]. According to the earlier publication reports, the red and blue spectral components are the major contributors to crop growth, such as promotes vegetative growth [16] and compactness [17,18] and creates aroma and nutritional value [19], as shown in Figure 1. While blue light has a short wavelength, it helps the plant adjust its growth [20] with the environmental interaction at a different stage and promotes early vegetative growth. Moreover, the blue component significantly affects the shoot architecture, resulting in a compact and dense plant [21], and increases the vegetative growth (leaves). In contrast, red light, promotes leaf elongation [22,23]. These wavelengths correlate to the five photosensory systems of a plant biosystem, namely, phytochromes (PHY), phototropins (PHOTO), cryptochrome (CRY), Zeitlupe (ZLT), and UVB-resistance locus 8 (UVR8). Each one of these photosensory systems triggers different morphological changes in plants. For example, phytochromes (PHY), whose unique photosensory properties can profoundly have a major role in governing plant elongation, flowering time, and leaf expansion [24], perceives light strongly in the red (660~700 nm) and far-red regions (700~750 nm) [25]. In contrast, the phototropin (PHOTO), cryptochrome (CRY), and ZLT system absorb light actively in the blue (400~495 nm) and UV-A (315~400 nm) regions [26], predominantly regulates plants hypocotyl elongation, and play an indispensable role in blue light facilitated stomatal opening [27,28] and controls the prosperity of an effective photoperiodic blossoming inducer [28], while the UVB-resistance locus 8 (UVR8) system perceives light intensely in the UV-B (280~315 nm) regions [29], and controls the biosynthesis-related genes expressions [30]. On the other hand, the far-red and UV ranges have secondary impacts on specific plants’ growth. In contrast, the green-yellow spectral components (sometimes called tertiary light due to their minimal impact) have a marginal role in the photosynthesis process. It is essential to mention that less research has been conducted to investigate the optimum LED illumination and its effect on water and electricity uses efficiency and the morphological development of sweet basil plants.

In addition to that, to increase the further crop yield, greenhouse designers are currently investigating the use of smart glass that enables effective light management by transmitting the solar spectral components that effectively contribute to the photosynthesis process while blocking the ineffective radiations, which typically lead to plant transpiration and photoinhibition.

The market for industrial sweet basil (*Ocimum basilicum* L.) for pesto, frozen, and dried markets are developing. Besides being popular as a spice, basil contains essential oils used in the medicine and chemical industry. Note that relatively little information exists regarding the effects of narrow-band lighting technologies on the physiological and morphological development of sweet basil and its resource use activity. However, several research groups have investigated the light spectrum’s effects on the yield of different greenhouse-grown vegetables and herbs [31,32]. Nevertheless, relatively few research groups have reported the impact of lighting outside of the well-known photosynthetically active radiation (PAR) wavelengths (400 nm to 700 nm), especially the effect of far-red wavelengths (~735 nm) on the physiological development of sweet basil plants. Therefore, this work aims to investigate the illumination spectrum that achieves significant biomass improvement of sweet basil plants through prior iterative refining of experimental circumstances and after a careful statistical analysis.

## 2. Materials and Methods

### 2.1. Plant Materials and Growing Conditions

The experiments were carried out during March 2020 in three separate grow tents at the Electron Science Research Institute (ESRI), Edith Cowan University (ECU), Australia.

A Heliospectra^TM^ LX602C tunable LED light source was hung inside in each grows tent above the plants at 1.26 m from the top of each pot. All pots were placed on the floor. The tunable LED sources are designed to cover a 2 m × 1.80 m area at a mounting height of 2 m. Therefore, they were adequate for illuminating the species inside the three 1.5 m × 1.5 m × 2.0 m grow tents. We used 60 plants for the experiments (20 plants per light treatment). After seedling transplanting, the pots were randomly distributed around the center of each tent over a circular area of a diameter ~0.75 m, and hence, the LED illumination was almost uniform for all pots. The sweet basil (*Ocimum basilicum* L.) seedlings were obtained from Bunnings warehouse, Joondalup, Western Australia, and transplanted individually into 125 mm (diameter) plastic pots with 650 gm of potting mix from Scotts Osmocote^®^ Plus Organics Vegetable and Herb Mix, Australia. The plants were watered regularly to maintain optimum soil moisture. We used a conventional soil moisture meter that indicates the amount of water needed to keep the soil moisture at the same level. After transplanting the seedlings, each pot was watered according to needs and kept as it is for one day to allow the water to soak in properly. To supply nutrients to the plants, a 10 mL of diluted liquid nutrient (Scotts Osmocote^®^, Melbourne, Australia) was mixed with 1490 mL of water. The resultant 1500 mL solution was split equally into 15,100 mL units, and each unit was supplied to a pot after every seven days from the start of LED illumination. Data (canopy temperature, air temperature, soil temperature, soil pH, humidity, LED consumption power, LED illumination uniformity) were collected at night.

### 2.2. Lighting Treatments

**Figure 2:**
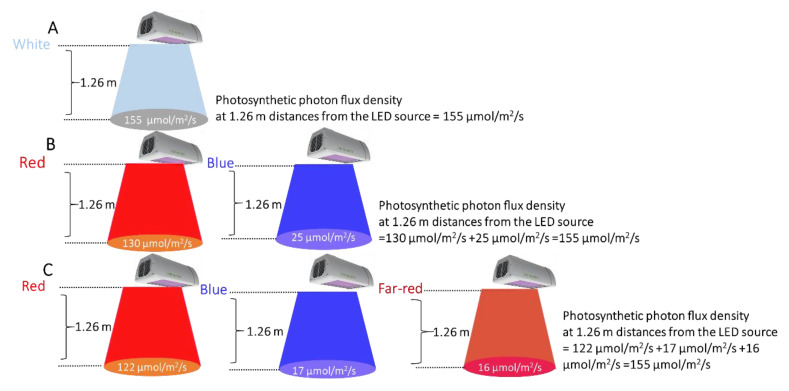
The photosynthetic photon flux densities (PPFD) of the Heliospectra^TM^ grow lights measured using a LaserCheck optical power meter, at different distances from the light-emitting diode (LED) source, for all tents.

Three different LED illumination spectra were applied in the grow tents, namely, W*, BR*, and BRF*. The power consumption was measured for each tent using an intelligent power meter (Electus Distribution Pty Ltd, Sydney, Australia). Note that the Heliospectra^TM^ LX602C tunable LED source enables the output power level of the individual LED spectral components to be systematically controlled between 0% and 100%, allowing a wide range of LED illumination spectra to be generated. The Heliospectra^TM^ LX602C LED sources were switched on every day from the afternoon (3:00 p.m.) to morning (11:00 a.m.). Thus, the illumination period was 20 h per day. Figure 2 shows the measured photosynthetic photon flux density (PPFD) at different distances from the LED source for all tents. As shown in Figure 2, the measured photosynthetic photon flux densities (PPFD) at 1.26 m from the LED sources (i.e., at the pot top-level) were 155 ± 1 µmol m^−2^ s^−1^ for all the tents. Note that the photosynthetic photon flux density is the sum of the flux densities corresponding to the individual LED colors used to generate the LED illumination spectrum [33]. The measured air temperature and relative humidity were in the 22 °C to 26 °C range and the 60~75% range, respectively, for all tents.

In order to accurately measure the LED intensity, we calibrated the LED power levels with a hand-held optical power meter (LaserCheck, Coherent Inc., Santa Clara, CA, USA), which has a power range of 10 µW to 1000 mW and an active aperture diameter of 8.0 mm and operates over the wavelength range 400 nm to 1064 nm. The power density (W/m^2^) at a specific distance from each light source was calculated by measuring the power output and dividing it by the active aperture area of the optical power meter. The average fresh mass (FW) of plants was measured using a high-sensitivity scientific laboratory balance (Westlab, Mitchell Park, Australia) with an accuracy of 0.01 g. Then, the leaves were placed in Kraft paper envelopes (27 cm × 22 cm) and heated in an oven (Furnace Technologies Pty Ltd, Jandakot, Australia) at 60 °C for 120 h until moisture was fully evaporated and a constant dry mass state was attained [34].

### 2.3. Measurements and Data Collection

**Figure 3:**
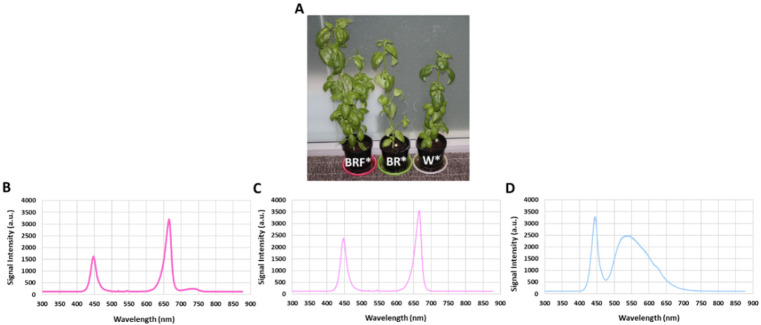
(**A**) Photos of typical sweet basil pots from the BRF*, BR*, and W* grow tents after 28 days of LED illumination. (**B**) BRF* illumination spectrum; (**C**) BR* illumination spectrum; and (**D**) W* illumination spectrum. W*—only white LED illumination; BR*— combination of red (R) and blue (B) LED illumination with relative R and B intensities of 84% and 16%, respectively); BRF*— combination of R, B and far-red (F) LED illumination, with relative R, B and far-red (F) intensities of 79%, 11%, and 10%, respectively.

After 28 days of LED illumination, morphological parameters of 24 out of 36 plants, namely, plant fresh mass (g), plant dry mass (g), plant height (cm), plant stem diameter (mm), leaf fresh mass (g), leaf dry mass (g), energy use efficiency (EUE) and water use efficiency (WUE) were measured. In order to measure the improvement in cultivation time for the BRF* illumination, the remaining 12 plants were kept in the white tent for two more weeks (14 days), and their morphological parameters were measured (i.e., on day 42). Note that the EUE, expressed as g FW kW^−1^, is defined as the ratio of the fresh mass of the sweet basil plant and the electricity consumption of LEDs, and the WUE, expressed in g FW L^−1^ H_2_O, is defined as the ratio between the leaf fresh mass and the volume of water used. Generally, WUE increases the plant’s fresh mass [35] and fruit yield [36]. During the experiments, the air temperature (T_air_ in °C) and relative humidity (RH%) inside each tent were measured using a thermometer (Green May International Ltd, Shenzhen, China), and data were manually logged on every day at 8:00 pm. The leaf temperatures were measured manually every day at 8:00 pm using an infrared thermometer whose laser beam probe was applied to the leaf surface area (Wiltronics Research Pty Ltd, Alfredton, Australia). At the end of the experiments, the average height of the sweet basil plants was measured and recorded using a tape measure (Stanley Tools, Melbourne, Australia) with 0.01 m precision. The average stem diameter of the sweet basil plants was measured using a digital Vernier caliper with an accuracy of 0.01 mm (Kincrome Australia Pty Ltd, Melbourn, Australia). The Heliospectra LED light sources are programmed to emit wavelengths of light at the broader spectrum white LED light (5700 K white visible light having peaked at approximately 446 nm, 534 nm, and 625 nm). As shown in Figure 3, the white LED spectrum contains 24% blue light, 58% green light, and 18% red light.

The soil moisture (dry to wet range only) and pH were measured using a ZD-07 4 in 1 digital soil pH meter (NDI Instrument and Hand Tools, Cheltenham, Australia) by inserting the probe of the instrument as vertically as possible and down halfway between the plant stem and the edge of the pot and taking several readings for averaging. Table 1 shows the measured PPFD, relative humidity, soil temperature, pH level, leaf temperature, and energy consumption for all grow tents.

### 2.4. Statistics

The data sets collected from the experiment were analyzed using the one-way analysis of variance (ANOVA) to find out if our experimental results are significant. Once we conducted the ANOVA test and found the test results statistically significant, at least two groups have significantly different means. Then, we intended to locate the specific difference and wanted to find out where our differences indeed came from. Therefore, to determine which groups are different from each other, we had to conduct a post hoc test. Descriptive statistical parameters, such as mean, standard deviation, and standard error (SE), were calculated using the IBM SPSS Statistics software package, version 27.

## 3. Results

### 3.1. Effect of Light Quality on Plant Growth and Morphology

**Figure 4:**
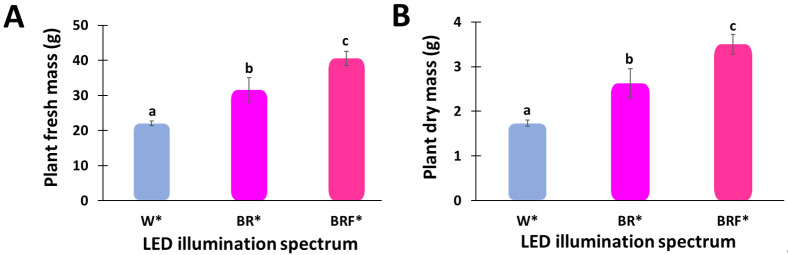
Effect of different LED illumination spectra on the growth, and development parameters, such as (**A**) fresh mass (g); and (**B**) dry mass of sweet basil (*Ocimum basilicum* L.) plants grown in 2 m^2^ tents. Different letters indicate a significant difference among the treatments, *p* ≤ 0.05.

In this study, the effects of blue, red, and far-red light spectra are mainly investigated. Results show that the plants’ growth is substantially different under the grow-tent environment, such as post hoc analyses revealed that the W*-illuminated tent produced the lowest average fresh biomass, 22.08 g, which was considerably lower than that produced by the BRF*-illuminated tent, 40.58 g, and the BR*-illuminated tent, 31.58 g, as shown in Figure 4A.

We have identified the best LED wavelength bands required to significantly improve the growth rate of sweet basil (*Ocimum basilicum* L.) plants in grow tent environments by optimizing the LED spectrum illuminating the plants. Post hoc comparisons using the Tukey’s–Kramer HSD tests indicated that the W*-illuminated tent produces the lowest mean dry biomass (M = 1.73 g; standard deviation, SD = 0.20 g), and this is substantially lower than that produced by the BRF*-illuminated tent (M = 3.49 g; SD = 0.62 g), and the BR*-illuminated tent (M = 2.62 g; SD = 0.93 g), as shown in Figure 4B.

### 3.2. Effect of Light Quality on Resource Usage Efficiency

**Figure 5:**
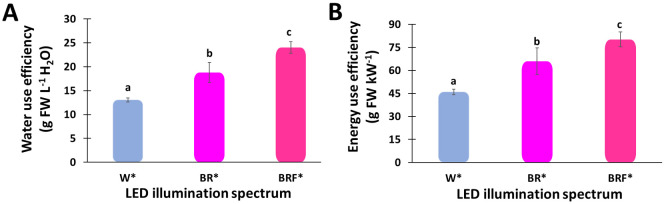
Effect of the different LED illumination spectra on the mean values of (**A**) water use efficiency (WUE) and (**B**) energy use efficiency (EUE) of sweet basil (*Ocimum basilicum* L.) plant grown in 2 m^2^ tents. Different letters indicate a significant difference among the treatments, *p* ≤ 0.05.

Figure 5A shows the WUE for the three LED illumination spectra. The WUE for the W*-, BR*- and BRF*-illuminated tents are 13 g FW L^−1^ H_2_O, 18 g FW L^−1^ H_2_O, and 24 g FW L^−1^ H_2_O.

Note that the uniformity of photosynthetic photon flux (PPF) is crucial for indoor plant growth. This prevents photons from being spread over, reducing energy consumption by improving their utilization inside the grow tent. The energy use efficiencies (EUE) is defined as the average biomass produced per unit of electrical energy used for plant illumination.

The energy use efficiencies (EUE) for the three LED illumination spectra are shown in Figure 5B, revealing that the EUE for the W*-, BR*- and BRF*-illuminated tents are 46 ± 1.7 g FW kW^−1^, 65 ± 8.6 g FW kW^−1^, and 80 ± 4.8 g FW kW^−1^. Here the BRF* spectrum yielded a significantly higher EUE than that of the W* spectrum.

### 3.3. Cultivation Cycle Improvement

**Figure 6:**
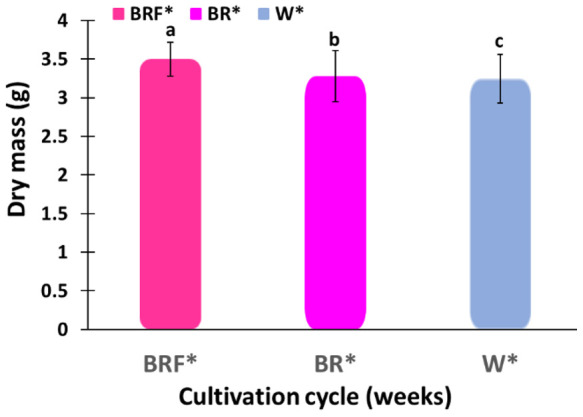
Biomasses of basil plants after 6 weeks in the W* illuminated tent and after 4 weeks in the BRF*-illuminated tents. Different letters indicate a significant difference among the treatments, *p* ≤ 0.05.

Figure 6 shows the biomasses of basil plants after 6 weeks in the W*-illuminated tent and after 4 weeks in the BRF*-illuminated tents. Note that, after 6 weeks, the average dry mass of the basil plants grows in the W*-illuminated tent was around 3.2455 g per plant, and that was 93% of the average dry mass per plant grown over a period of 4 weeks in the BRF*-illuminated tent. Note that more than 60% of the basil plants grown in the W*-illuminated tent bolted after 6 weeks, whereas none of the basic plants showed bolting after 4 weeks. These results demonstrate the ability of the BRF* illumination spectrum to (i) achieve high crop yield and (ii) high crop quality (no bolting).

## 4. Discussion

The spectral components of LED illumination significantly affect both plant physiology and growth morphology [37,38]. The visible light is typically the major contributor to the photosynthesis process for sweet basil plants, and by incorporating LED supplemental lighting in indoor farming, the plant growth rate can be increased significantly [39,40]. In the present study, crop yield enhancement was associated with a higher fraction of far-red light, a phenomenon that both Lee et al. [41] and Murchie et al. [42] have reported. According to the Emerson effect, both the red and far-red bands are significant contributors to the photosynthetic process for plants [43]. Thus, adding far-red LED illumination typically increases morphological parameters of indoor-grown plants. For example, increasing the ratio of the red intensity to far-red intensity increases the leaf length [44] and yield [45]. Sometimes, when the plant sizes become large, the degree of shading in the greenhouse becomes high, and plants tend to modulate their growth to resume a light-seeking strategy [2]. This is referred to as the shade avoidance syndrome and is, in effect, partial etiolation. Shade avoidance enables a plant to anticipate future competition for light by reducing reliance on resources for branching and capitalizing more on height growth [3]. Shade avoidance also causes an altered partitioning of photosynthate in favor of vegetative tissues, which can decrease yield in seed-producing crops [4]. The shade-avoidance response can be induced under other conditions such as crowding and under different conditions that cause a reduced ratio of red to far-red light, indicating phytochromes’ involvement [4].

**Figure 7:**
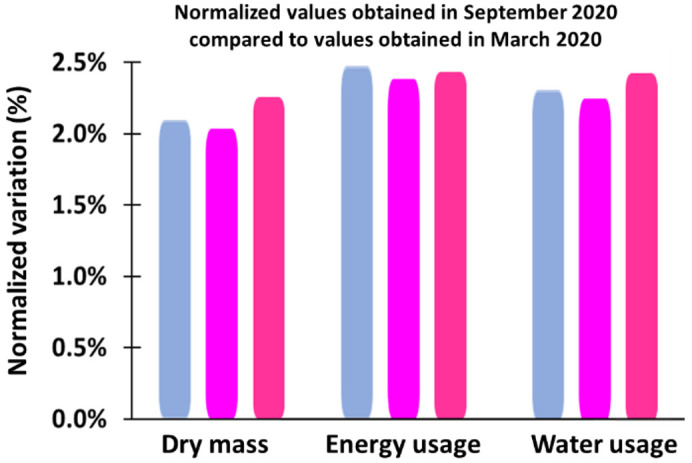
The normalized values obtained in the first experiment in September 2020 compared to values obtained in the second experiment in March 2020.

Note that, in order to validate these results, another set of experiments was conducted in September 2020, and the variances in plant yield, water use efficiency, and energy use efficiency were less than ±2.5%, as shown in Figure 7.

The inside walls of each grow tent used in the experiments were coated with silver reflective coatings to prevent shading. Shading typically results in plant “bolting” and reduces the oil content, and hence, the biomass, in the basil leaves [46,47]. Note that too much light would also reduce the plant biomass as a result of plant tip burns. Sweet Basil (*Ocimum basilicum* L.) plants were specially selected for this study since they can be grown in a protected environment with higher temperatures in a long-day growth mode. Note that, after 6 weeks, the average biomass of the sweet basil plants grown in the W*-illuminated tent was around 3.2455 g per plant, and that was 93% of the average biomass per plant produced over a period of 4 weeks in the BRF*-illuminated tent. Also note that, in this experiment, more than 60% of the basil plants grown in the W*-illuminated tent bolted after 6 weeks, whereas none of the basic plants showed bolting after 4 weeks. Therefore, by better understanding the effects of lighting conditions (including the effect of the length of the day and night illumination) on the vegetative growth and reproductive growth (bolting, also known as preliminary flowering), the plant yields can be improved significantly.

Furthermore, in the experiments, it was observed that increasing the red LED intensity inhibits the transition to flowering (i.e., bolting) in basil plants, and this also has been observed recently [48]. It was shown that compared to 100% red LED illumination, the combination of red and blue LED illumination sources (91% red + 9% blue, by the photon counts) has a positive impact on sweet basil, spinach, and lettuce, in terms of biomass, plant height and leaf size [49]. Moreover, it was reported that the highest shoot dry mass of sweet basil (*Ocimum basilicum* L.) plants is attained with R70B30 LED illumination (70% red + 30% blue light with 250 ± 10 µmol m^−2^ s^−1^) [50]. Note that the phytochrome (PHY) photoreceptors of sweet basil absorb light strongly in the red (660~700 nm) and far-red regions (700~750 nm) [25], and this enhances the vegetative development (biomass) as well as architectural development of the plant [51]. In the experiments, a similar kind of results was achieved. For example, BRF*-illuminated tent produced a higher dry mass, 3.49 g, after 4 weeks, which was considerably higher than the average dry mass of 1.73 g produced by the W*-illuminated tent at the same time.

The basil’s WUE values are 3 g FW L^−1^ H_2_O in open field cultivation, while 20 to 22 g FW L^−1^ H_2_O are observed in potted grown basil in European climate conditions [52]. Similar results were also found in the present experiments, such as higher biomass were produced with lower water consumption, i.e., the WUE increased to above 24 g FW L^−1^ H_2_O in BRF*-illuminated tent (Figure 5A) [53]. Note that, in the present study, the WUE in the BRF*-illuminated tent was increased by 83% and 27%, respectively, compared to the W*-illumination (Figure 4A) and the BR*-illumination. In contrast, the WUE for BR*-illuminated tent was improved by 47% compared to the W*-illuminated tent. This is due to the higher red portion of the LED spectrum. While the red part of the LED spectrum increased, the quantum efficiency of the photosynthesis process decreased; however, the transpiration decreased more rapidly, resulting in increased water usage efficiency [54]. This increased water usage efficiency could also be associated with changes in the stomatal behavior of the plant. Typically, the soil temperature, which also affects basil plants’ growth rate, depends on the ratio of the energy absorbed by the soil to the energy lost from the soil. Note that the soil temperature affects the soil moisture because high soil temperature leads to water evaporation and crop transpiration. Hence, to maintain a high plant growth rate, the amount of water supplied to the plants must be continuously monitored and optimized. The basil plants illuminated by the W* spectrum exhibited a higher leaf surface temperature than the plants illuminated by the BR* and BRF* spectra (Table 1). This could be attributed to the non-photosynthetic spectral components being absorbed by the crop and converted to heat. Note that the leaf surface temperature is typically affected by illumination, relative humidity and ambient temperature. When a photon of light hits the plant leaf, it can either be reflected, transmitted, or absorbed. The photons that participate in the photosynthesis process (e.g., blue, red, and far-red photons) typically have less impact on the leaf temperature than the photons absorbed by the plant but do not contribute to photosynthesis (e.g., UV, green, and IR photons). Therefore, measuring the leaf surface temperature under light illumination is an indirect indication of the effectiveness of the illumination spectrum on the photosynthesis process, thus energy usage efficiency. In the experiments, based on the basil yield, it should be noted that the EUE was greater when higher spectral portions were allocated to the red region as for the case of the BRF* illumination (80 ± 4.8 g FW kW^−1^) and the BR* illumination (65 ± 8.6 g FW kW^−1^) (Figure 4B), due to a larger yield increase observed in these treatments, compared to the W*-illumination (46 ± 1.7 g FW kW^−1^).

## 5. Conclusions

We have experimentally investigated the effect of LED illumination spectra on the growth of sweet basil plants. Specifically, the plant fresh mass (g), plant dry mass (g), energy use efficiency (EUE), water use efficiency (WUE), and plant cultivation cycle were measured for sweet basil plants grown in three different grow tents illuminated with (i) white (W*), (ii) blue (B) and red (R); and (iii) blue (B), red (R) and far-red (F) LED spectra. Post hoc analyses have revealed that the BRF*- and BR*-illuminated tents produced, respectively, 83% and 42% higher average fresh biomass than that produced in the W*-illuminated tent. For the average dry mass, results have shown that the BRF*- and BR*-illuminated tents produced, respectively, 100% and 51% higher average dry biomass than that produced in the W*-illuminated tent. Results have also shown that, after 6 weeks, the average biomass of the basil plants grown in the W*-illuminated tent is 93% of the average biomass per plant grown over a period of 4 weeks in the BRF*-illuminated tent, and that, more than 60% of the basil plants grown in the W*-illuminated tent bolted after 6 weeks, whereas the basil plants were bolting-free after 4 weeks. These results have demonstrated that the BRF*-illumination treatment enables higher crop yield and quality (no bolting) in comparison with the W*-illumination treatment.

In addition, experimental results have shown that the water usage efficiency with the BRF* spectrum was 24 g FW L^−1^ H_2_O, compared to 13 g FW L^−1^ H_2_O, and 18 g FW L^−1^ H_2_O for the W*- and BR*-spectra, respectively. Moreover, results have revealed that the electricity usage efficiency with the BRF* spectrum was 80 ± 4.8 g FW kW^−1^, compared to 46 ± 1.7 g FW kW^−1^ and 65 ± 8.6 g FW kW^−1^ for the W* and BR* spectra, respectively. Therefore, the results of this study have demonstrated the commercial viability of both BRF*-, and BR*-illuminated grow tents compared to the commonly used W*-illuminated counterparts.

The protected environment results presented in this work paves the way towards the development of glass greenhouses employing spectrally selective optical coatings that pass the optimum solar spectral components through, which significantly increase the yield of sweet basil.

## Figures and Tables

**Table 1:** The power being consumed (kWh, total per entire growth period per growth tent) and growing conditions applied to the cultivation of sweet basil plants in the grow tent.

Light Types	PPFD (µmol m^−2^ s^−1^)	Relative Humidity (%)	Soil Temperature (°C)	pH	Leaf Temperature (°C)	Energy Consumption (kWh)	Plant Height (cm)	Plant Stem Diameter (mm)	Leaf Fresh Mass (g)	Leaf Dry Mass (g)
W*	155 ± 1	64 ± 2	24.88 ± 1	6.20	22.78 ± 1	133 ± 1	383.75	3.79	11.04	1.45
BR*	155 ± 1	64 ± 2	24.98 ± 1	6.35	22.57 ± 1	133 ± 1	416.87	3.77	15.83	2.97
BRF*	155 ± 1	64 ± 2	25.19 ± 1	6.30	23.34 ± 1	133 ± 1	464.37	4.09	19.25	4.62

## Data Availability

The datasets generated and/or analysed during this study are available from the corresponding author on reasonable request.
